# Percutaneous deep venous arterialization with balloon angioplasty salvaged a life‐threatening critical limb in a hemodialysis patient after repeated pedal angioplasty failed: A case report

**DOI:** 10.1002/ccr3.7589

**Published:** 2023-06-20

**Authors:** Tomonari Takagi, Akira Miyamoto, Norihiko Ohura, Yasutaka Yamauchi

**Affiliations:** ^1^ Cardiovascular Center Takatsu General Hospital Kanagawa Japan; ^2^ Department of Plastic and Reconstructive Surgery Kyorin University Hospital Mitaka Japan

**Keywords:** chronic limb threatening ischemia, deep venous arterialization, end‐stage renal failure, limb salvage

## Abstract

Chronic limb‐threatening ischemia (CLTI) with severe below‐the‐ankle (BTA) lesions is often difficult to revascularize with endovascular treatment (EVT) and surgical treatment. We present a case of successful limb salvage using percutaneous deep venous arterialization (pDVA) in a patient with CLTI whose BTA lesion reconstruction failed. A 57‐year‐old man with diabetes mellitus and end‐stage renal failure on maintenance hemodialysis was referred to our hospital because of gangrene in the second and third toes of his left foot. EVT was repeated for the anterior tibial artery, posterior tibial artery (PTA), dorsal foot artery, and plantar artery lesions; however, revascularization below the ankle was unsuccessful. As the infection had spread to the sole of the foot, below‐the‐knee amputation was indicated, but the patient refused. Therefore, we performed pDVA on the left PTA simultaneously with a Lisfranc amputation. An arteriovenous fistula was created at the ankle joint using a Venous Arterialization Simplified Technique and a guidewire was inserted into the plantar vein. Balloon dilatation from PTA to the plantar vein was performed to complete the pDVA. Although repeated EVT was required to maintain blood flow in the pDVA, skin grafting was performed 3 months after the pDVA, the wound completely healed, and he was discharged 6 months after the DVA. The pDVA can be an option for limb salvage in patients with no‐option CLTI who are confronted by imminent amputation.

## Graphical abstract

Clinical course of wound healing following percutaneous deep venous arterialization.

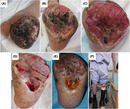

## 1 INTRODUCTION

Chronic limb‐threatening ischemia (CLTI) is a clinical syndrome in combination with rest pain, unhealed ulceration, and gangrene derived from diminished peripheral arterial circulation.^1^ In recent years, the number of patients with CLTI has been increasing due to the aging society and the rise in diabetes occurrence. In particular, end‐stage renal failure patients with CLTI are at high risk of major amputation because they frequently have severe below‐the‐ankle (BTA) lesions, such as type 3 in the Kawarada classification^2^ that are difficult to revascularize.

Recently, the efficacy of percutaneous deep venous arterialization (pDVA) has been reported for CLTI with severe BTA lesions that cannot be revascularized, known as the “no‐option CLTI.” In Europe and the United States, the LimFlow system is available as a dedicated device for pDVA, and its efficacy and safety have been reported in the PROMISE I study.^3^ However, the LimFlow system has not yet been approved in Japan, and we attempted to perform pDVA using balloon angioplasty.

## 2 CASE PRESENTATION

**FIGURE 1:**
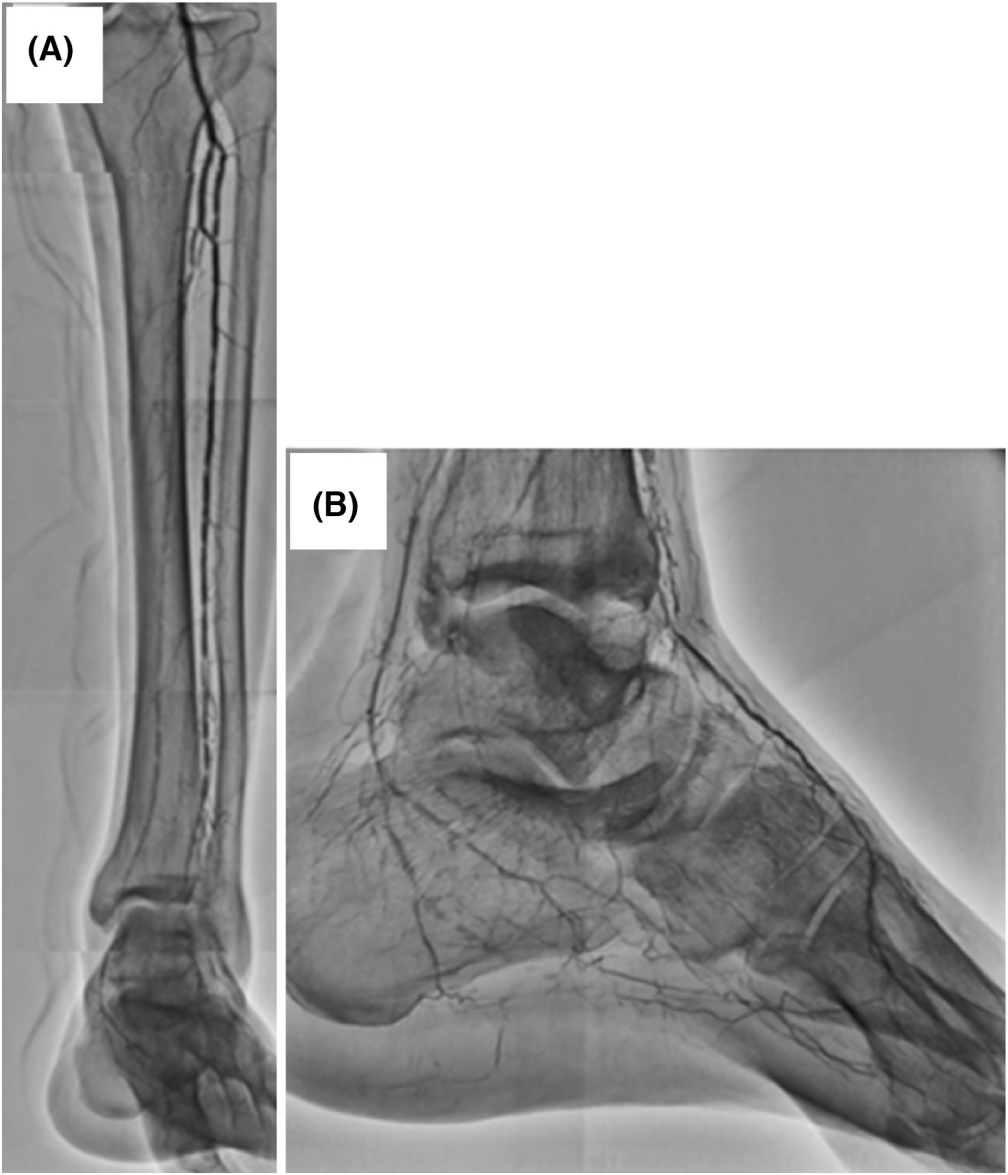
(A) Angiography show heavily calcified severe diffuse stenosis of the left anterior and posterior tibial arteries and occlusion of the peroneal artery. (B) Angiography show heavily calcified severe stenosis of the left dorsalis pedis and the medial plantar arteries and occlusion of the left plantar artery.

**FIGURE 2:**
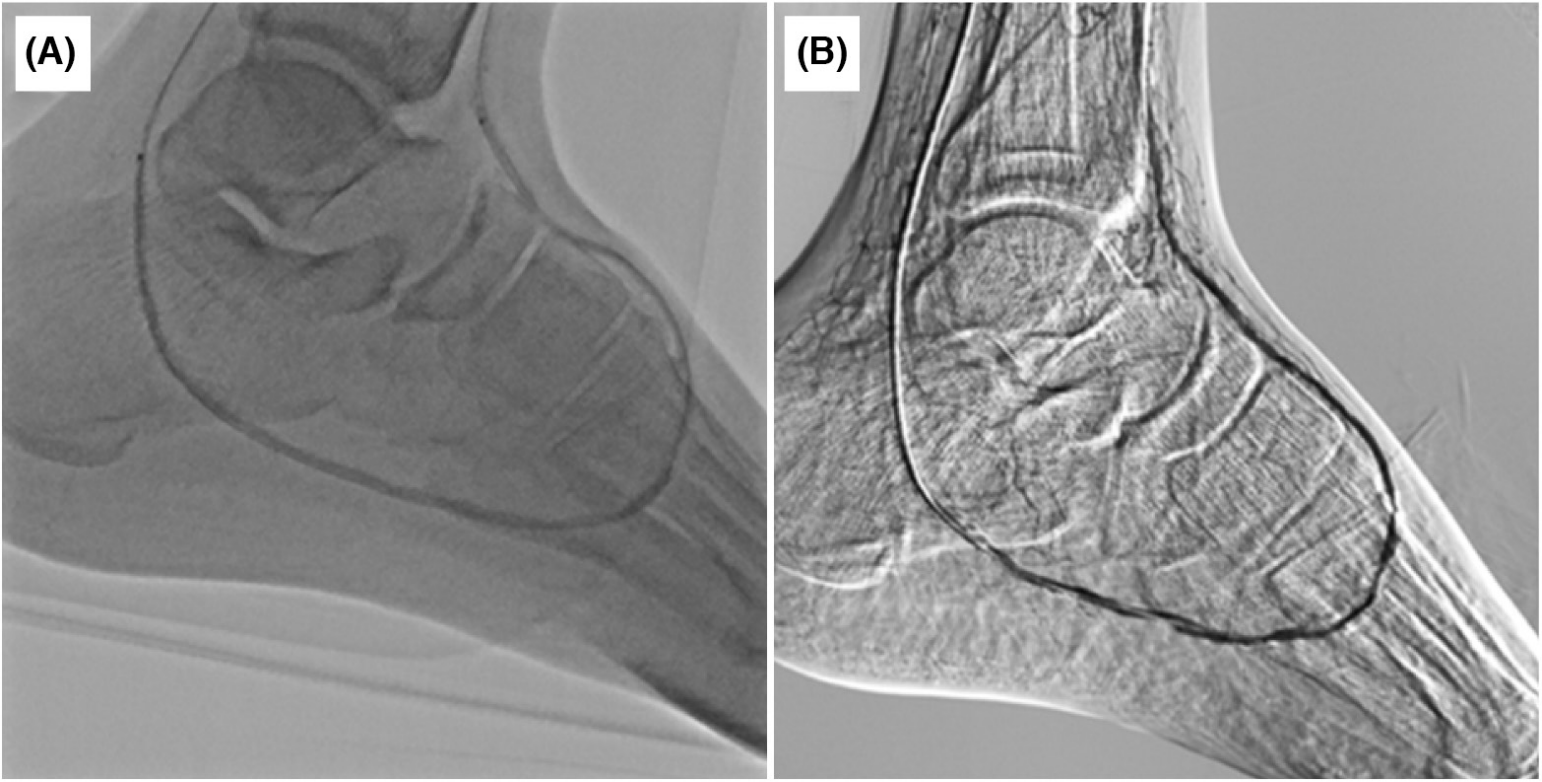
(A) Balloon dilation from the dorsalis pedis to the lateral plantar arteries using 2.5 × 180 mm balloon. (B) Angiography show patency of the loop of the dorsalis pedis to the lateral plantar arteries.

**FIGURE 3:**
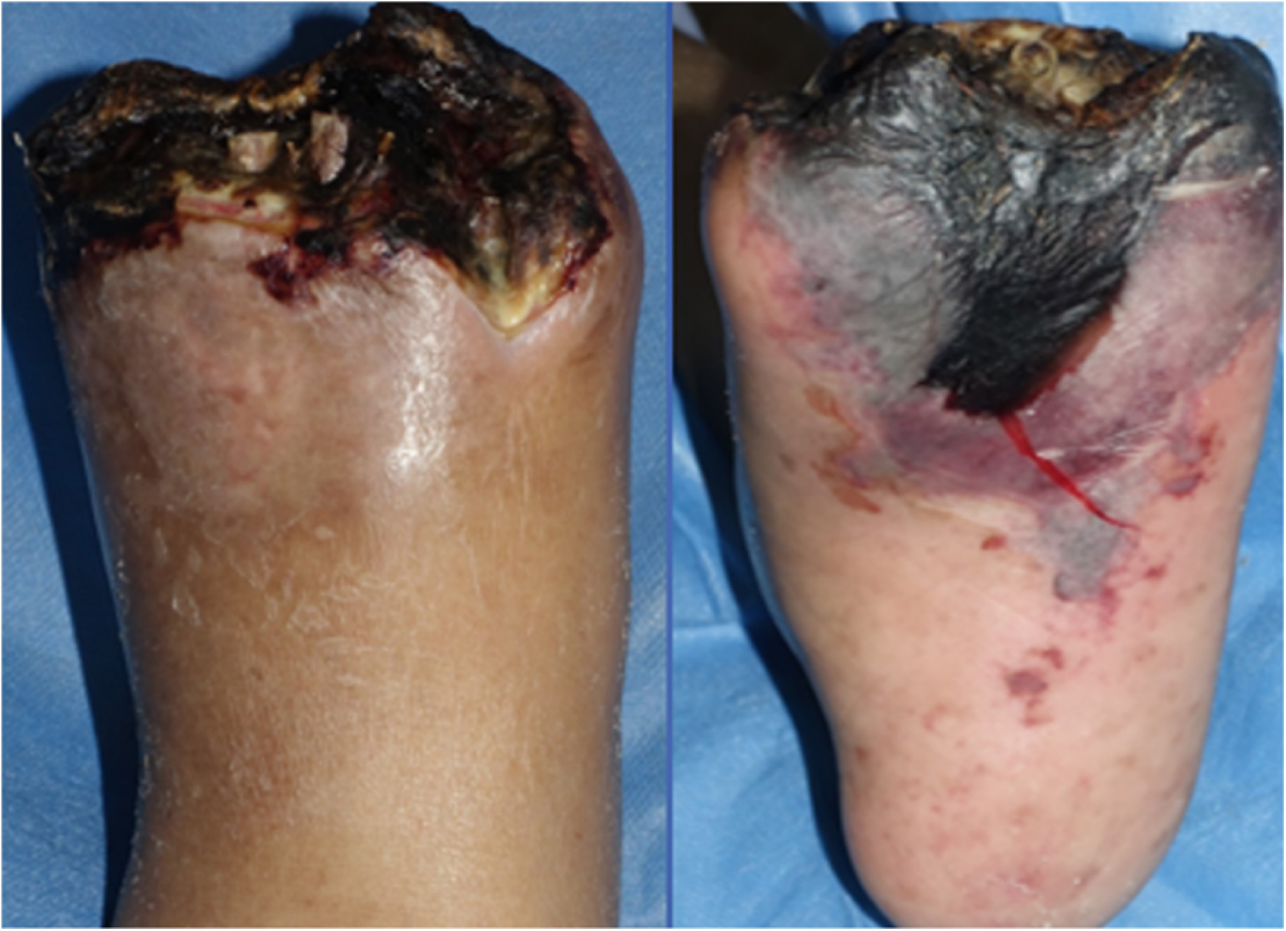
Gangrene quickly extended even after trans‐metatarsal amputation, and infection spread to the plantar region.

A 57‐year‐old Japanese man with a 25‐year history of maintenance hemodialysis and 37‐year history of type 2 diabetes mellitus was referred to our hospital for gangrene of the second and third toes of the left foot. Angiography revealed severe stenosis and occluded lesions with heavy calcification of the left below‐the‐knee (BTK) and BTA (Figure 1A,B). Repeated endovascular treatment (EVT) was performed on the BTK and BTA lesions to maintain pedal arterial flow. Eventually, the loss of distal flow in the pedal arch (Figure 2A,B) and spread of infection to the sole (Figure 3) implied the need for a BTK amputation. However, the patient refused to undergo BTK amputation; therefore, we attempted pDVA from the posterior tibial artery (PTA) to the planter vein simultaneously by performing the Lisfranc amputation.

## 3 PERCUTANEOUS DVA PROCEDURE

**FIGURE 4:**
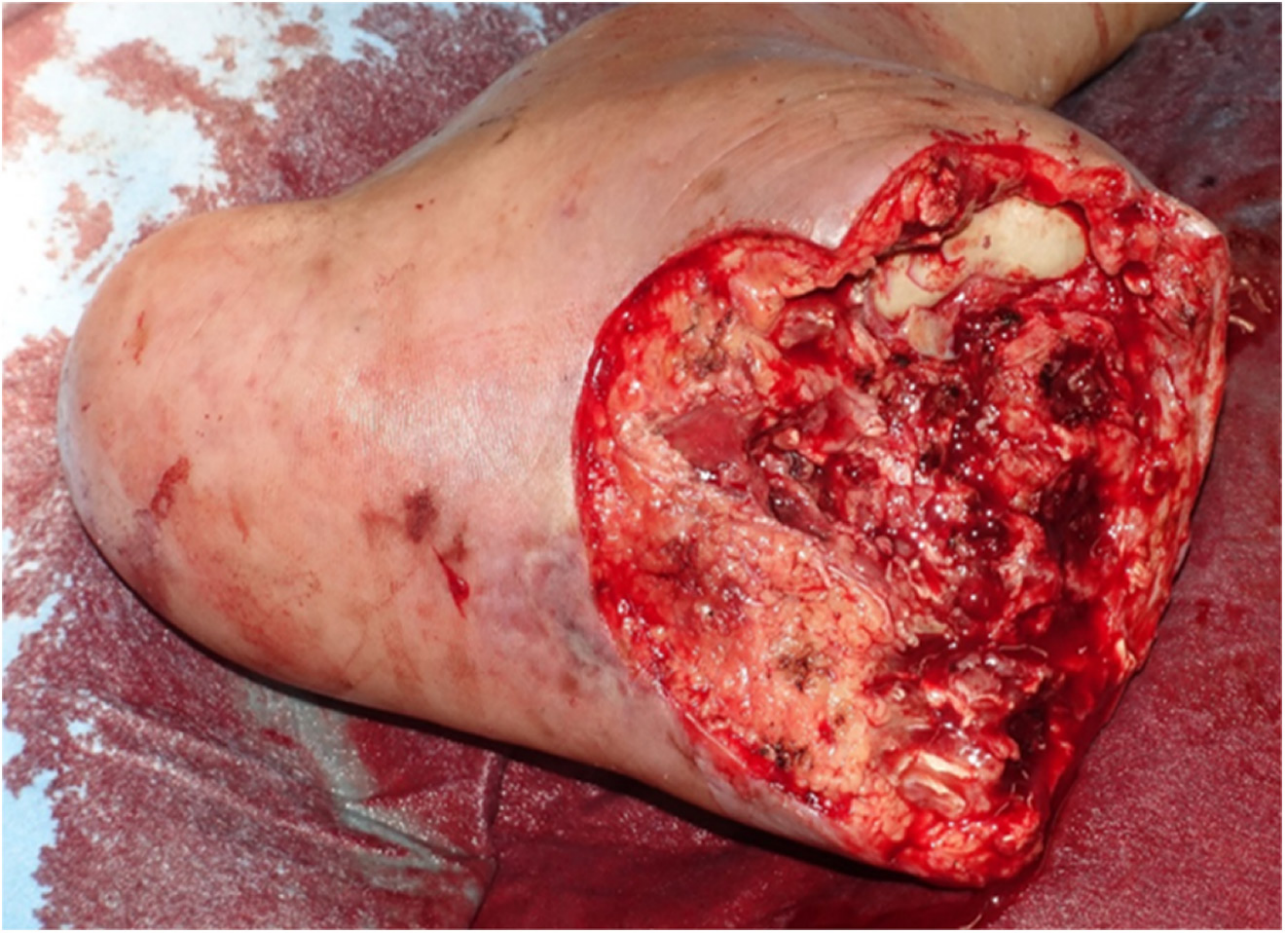
Lisfranc amputation was performed prior to percutaneous deep venous arterialization (pVDA).

After Lisfranc amputation (Figure 4), a 4.5Fr Parent Plus 53 cm (Medikit, Miyazaki, Japan) guiding sheath was inserted antegradely from the left femoral artery.

**FIGURE 5:**
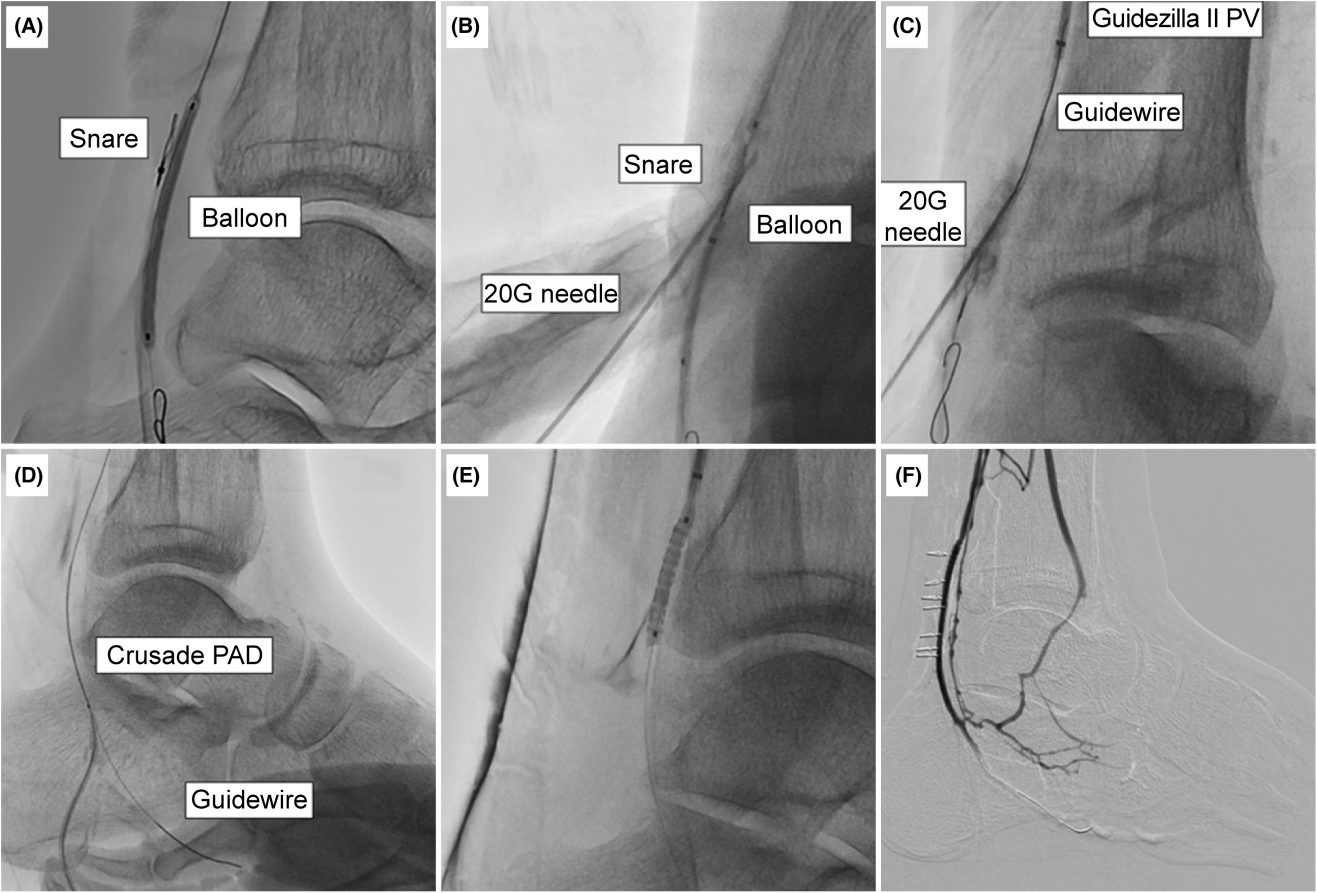
(A) The snare and balloon show an overlap under the fluoroscopic guidance. (B) The 22 G needle was inserted into the intra‐arterial balloon through the intra‐venous snare loop. (C) The guidewire (GW) is advanced into the intra‐arterial extension catheter from the 20 G needle. (D) The dual‐lumen catheter is advanced over the externalized GW, while the other GW is advanced into the lateral plantar vein. (E) The expanded polytetrafluoroethylene (ePTFE)‐covered stent was deployed to achieve hemostasis of the venous cut‐down site. (F) Angiography show distal deep venous system after pDVA.

The occluded PTA was dilated to the level of the ankle joint with a 2.5 × 40 mm JADE balloon (OrbusNeich, Shenzhen, China), and a guide extension catheter (Guidezilla II PV; Boston Scientific, MN, USA) was introduced distal to the PTA. Surgical cannulation of the posterior tibial vein (PTV) in the ankle was unsuccessful. Therefore, the PTV was punctured as distal as possible at the ankle joint under extravascular ultrasound guidance, and a slender 4Fr sheath (Prelude Ideal; MERIT MEDICAL, UT, USA) was antegradely inserted. A snare (4‐8 mm Atrieve Vascular Snare Kit; ARGON MEDICAL DEVICES, TX, USA) was introduced from the PTV to the point where it overlapped the inflated balloon at the distal PTA (Figure 5A), and a 20 G needle was used to penetrate the snare and the inflated balloon, in that order, from the body surface, under fluoroscopy (Figure 5B). A 0.014 inch guidewire (Gladius; Asahi Intec, Aichi, Japan) inserted from the needle was introduced into the PTA along the burst balloon and finally pulled out of the body through the guide extension catheter (Figure 5C). Using a CRUSADE PAD dual‐lumen catheter (Kaneka Medical, Osaka, Japan) introduced into the distal PTA, another 0.014″ guidewire (JUPITER FC 300 cm; Boston Scientific, Aichi, Japan) was grasped with a snare inserted into the PTV and passed into the PTV sheath. The CRUSADE PAD was introduced into the PTV through this guidewire and a new guidewire was introduced into the lateral plantar vein (Figure 5D). An arteriovenous fistula (AVF) was created by dilating a 3.0 × 240 mm JADE balloon (OrbusNeich, Shenzhen, China) from the lateral plantar vein to the PTA, while hemostasis was achieved at the needle puncture site and sheath insertion site of the PTV. However, balloon dilation failed to achieve hemostasis at the surgical cannulation site, and therefore a 2.8 × 19 mm coronary covered stent (GLAFTMASTER; Abbot, CA, USA) was placed to stop the bleeding (Figure 5E). Final angiography showed good blood flow from the PTA to the pedal veins; however, the lateral plantar vein was not contrasted because the pedal venous arch was amputated (Figure 5F).

## 4 CLINICAL COURSE AFTER pDVA

**FIGURE 6:**
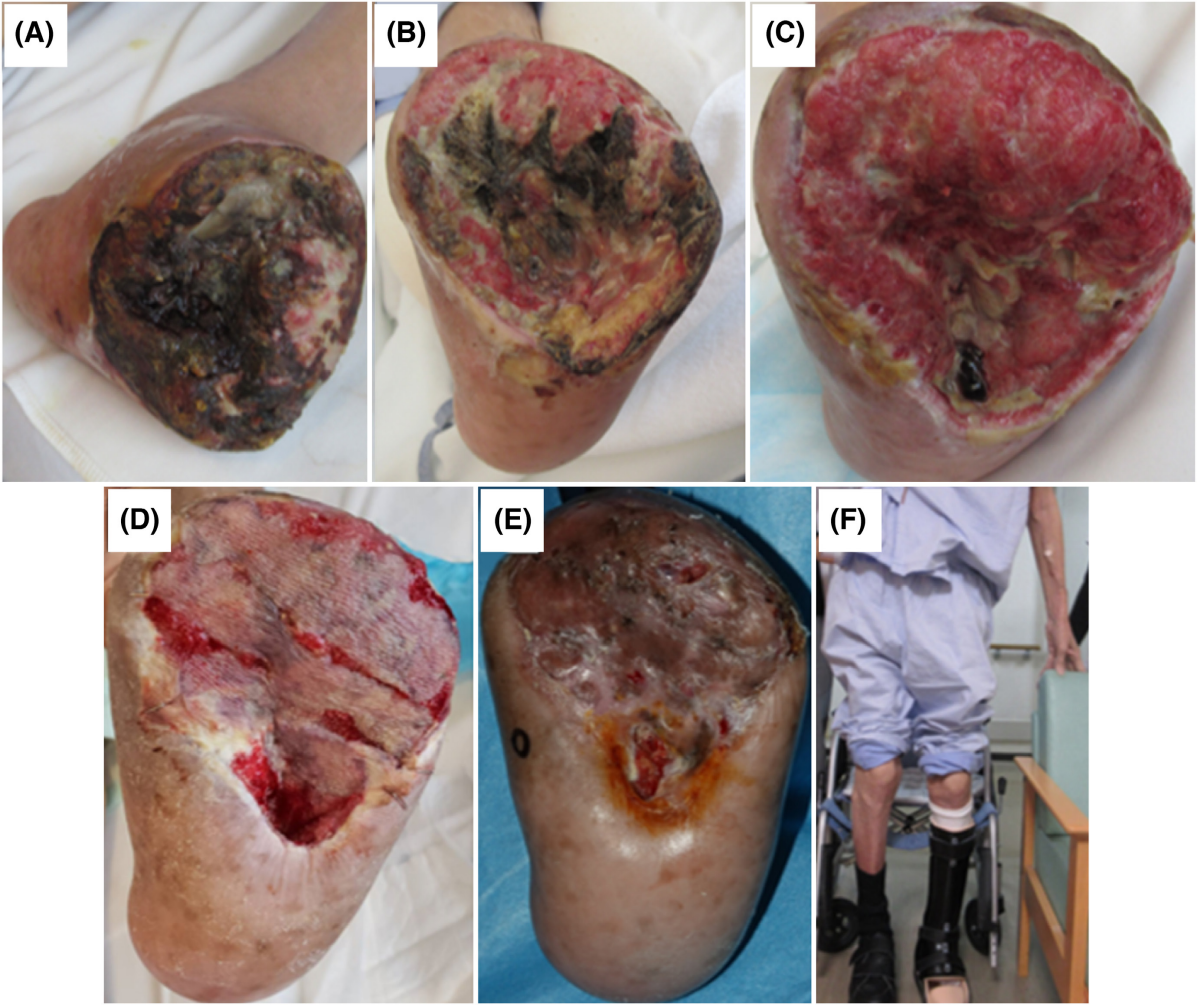
(A) Gangrene extended transiently after percutaneous deep venous arterialization (pDVA). (B) Granulation was gradually observed about 1 month later. (C) Then growth of granulation tissue promoted. (D) Skin grafting was performed about 3 months later. € Wound has almost completely healed. (F) He was discharged about 6 months later.

**FIGURE 7:**
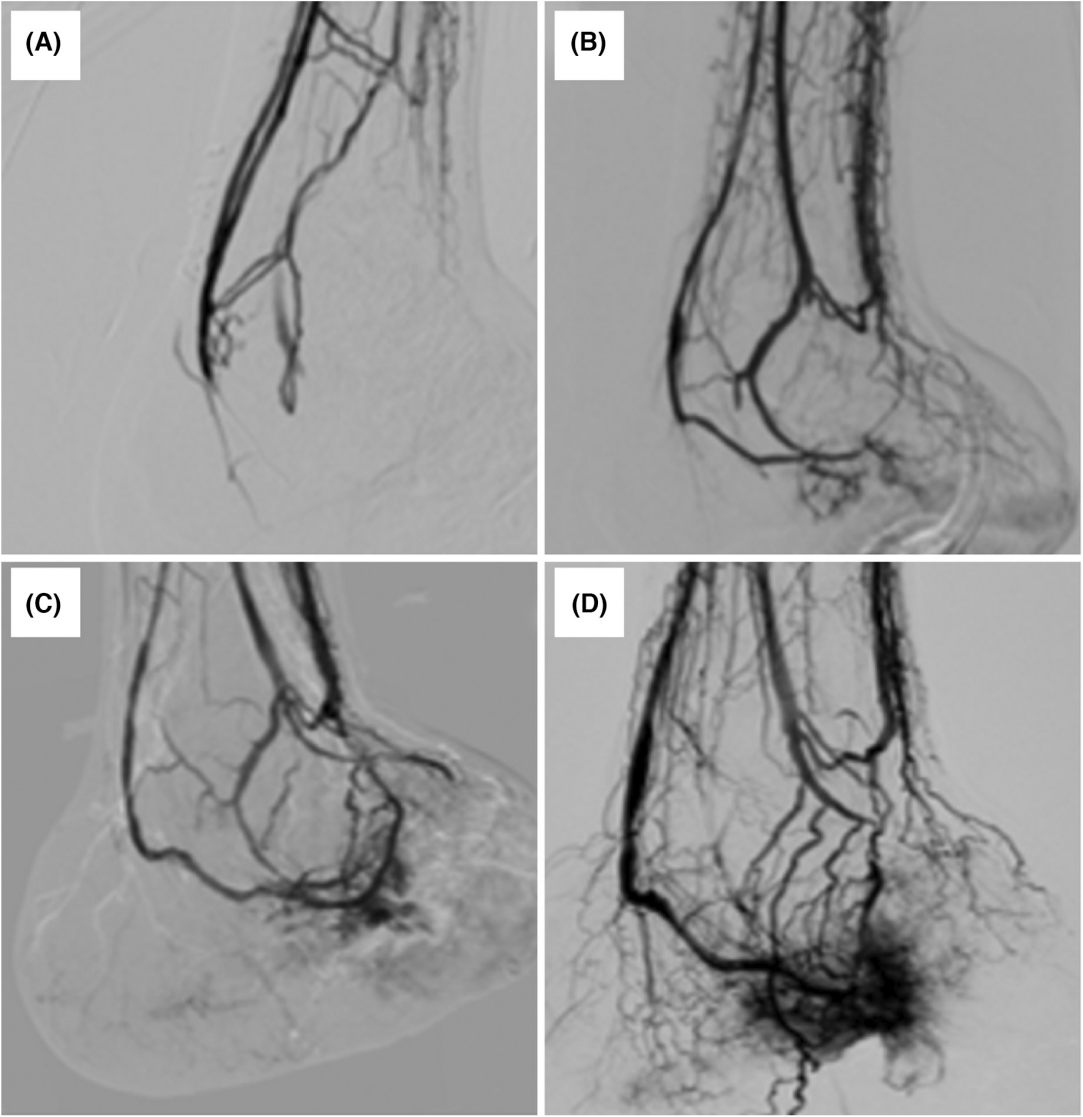
(A) Perfusion of the plantar remained poor about 1 month later. (B,C) Perfusion of the plantar gradually increased and wound blush is observed. (D) Striking collateral circulation is seen mainly on the plantar before discharge.

Necrotic tissue on the amputation surface increased up to 2 weeks after the pDVA (Figure 6A). Thereafter, a gradual increase in granulation was observed (Figure 6B). One month later, re‐EVT was performed because of covered stent restenosis; however, the pedal arteriovenous circuit was still poor (Figure 7A). EVT for stenosis or occlusion in the route of the pDVA was then repeated approximately every month, and the pedal arteriovenous circuit gradually improved (Figure 7B). Three months later, good wound brushing was observed on the foot (Figure 7C). Five months later, angiography revealed prominent collateral vessels mainly on the plantar side (Figure 7D). The wound was covered with good granulation tissue, and skin grafting was performed 3 months after pDVA (Figure 6C,D). Six months later, the wound completely healed, and he was discharged after being able to walk independently with a lower‐limb orthosis fabrication (Figure 6E,F).

## 5 DISCUSSION

Patients with CLTI frequently have BTK lesions, for which revascularization is important to achieve blood flow to the foot wound. Patients with CLTI undergoing hemodialysis are often complicated by severe BTA lesions. The clinical benefit of BTK angioplasty in patients undergoing hemodialysis is limited owing to severe BTA lesions, with hemodynamic improvement achieved in only half of the patients.^4^ In this case, EVT was repeated for BTK and BTA lesions, ultimately resulting in the loss of blood supply to the distal pedal arteries and Lisfranc amputation. Further, infra‐popliteal EVT was abandoned because of the lack of distal BTK outflow. Recently, the efficacy of pDVA for no‐option CLTI has been reported.^5^, ^6^ DVA is a technique used to reverse venous flow and supply arterial blood to capillaries and ischemic pedal tissues to relieve rest pain and promote the healing of chronic wounds. The LimFlow system is currently the only dedicated device capable of performing pDVA, and its efficacy and safety have been reported in the ALPS multicenter study registry^7^ and PROMISE 1 study.^3^ However, a LimFlow system is not yet available in Japan. Gandini et al. reported the efficacy of distal plantar venous arterialization with conventional balloon angioplasty for no‐option CLTI.^8^ They performed reentry from the distal PTA to the plantar vein with unidirectional wiring under fluoroscopy, with success in seven of the nine cases. This method involves the possibility of wire manipulation because the plantar vein cannot be seen on arteriography. Therefore, we attempted wire reentry to the distal PTV at the ankle joint using the Venous Arterialization Simplified Technique (VAST)^9^ reported by Ysa et al. to perform the pDVA with a conventional balloon angioplasty. The lateral plantar vein was not contrasted because it was already ligated at the distal portion; however, good flow was observed from the PTA to the pedal veins via the medial plantar vein. It has been reported that a new arteriovenous circuit created by pDVA takes 6 weeks to mature, during which time the pDVA must be kept open.^10^ In fact, in our case, while maintaining the patency of the pDVA, granulation regenerated after 2 weeks. After 3 months, the wound had improved enough to allow for skin grafting, and angiography showed angiogenesis around the wound. However, pDVA restenosis frequently occurs early, even with Limflow, and strict follow‐up with DUPLEX should be performed every 2 weeks for the first 2 months. In our case, EVT was performed for restenosis of the AVF and re‐occlusion of the recanalized PTA every month within 3 months after pDVA. There are certain limitations associated with this method. The technical problem with our pDVA method is that, unlike pDVA with LimFlow, the occluded PTA must be recanalized by balloon angioplasty below the ankle joint to create the AVF as distally as possible to minimize blood flow stealing centrally from the PTV branches caused by venous return. Venous valves are typically present in the planter veins and pedal venous arch, and valvotomy is often necessary to ensure blood flow to the pedal venous arch. We frequently employ cutting balloons or oversized balloons for valvotomy. In addition, our method implies a high risk of reocclusion because the long occluded PTA and AVF were dilated with a balloon alone without covered stents. However, our method has several advantages. It can be performed with reimbursed devices, and there is a lower risk of infection because no foreign material is used, the branches of the recanalized occluded PTA can be preserved, and there are no restrictions on future treatment due to stent graft placement. However, increased shunt flow through the AVF may lead to leg congestion and heart failure. Our patient had leg congestion because the pedal venous pressure might not have been elevated by the appropriate escape of shunt flow from the PTV branches to the central region. The blood flow rate of PTA was less than 200 mL/s, which is a very small shunt volume compared to that of an AVF for hemodialysis and is unlikely to be a cause of cardiac failure. One year after pDVA, our patient unfortunately had DVA occlusion, but no recurrence of ulcer was observed; it was assumed that angiogenesis by DVA created a collateral pathway to the foot. This method may be an effective means of saving the limb with no‐option CLTI.

## 6 CONCLUSION

Percutaneous DVA with balloon angioplasty without the LimFlow system was effective in limb salvage after repeat pedal angioplasty failed in patients with CLTI on hemodialysis.

## AUTHOR CONTRIBUTIONS

**Tomonari Takagi:** Conceptualization; investigation; visualization; writing – original draft. **Akira Miyamoto:** Supervision; writing – review and editing. **Norihiko Ohura:** Writing – review and editing. **Yamauchi Yasutaka:** Writing – review and editing.

## FUNDING INFORMATION

This study was not supported by any funding.

## CONFLICT OF INTEREST STATEMENT

The authors declare that they have no competing interests.

## ETHICS STATEMENT

The study was performed in accordance with the Declaration of Helsinki and approved by the ethics committee of our center.

## CONSENT

Written informed consent was obtained from this patient.

## PERMISSION TO REPRODUCE MATERIAL FROM OTHER SOURCES

All the materials are original, and we did not need permission to reproduce material from other sources.

## Data Availability

Data sharing is not applicable to this article as no new data were created or analyzed in this study.
